# Longitudinal pattern of resource utilization by aquatic consumers along a disturbed subtropical urban river: Estimating the relative contribution of resources with stable isotope analysis

**DOI:** 10.1002/ece3.8304

**Published:** 2021-11-11

**Authors:** Sai Wang, Tuan‐Tuan Wang, Wen‐Tong Xia, Zhong‐Bing Chen, Simon D. Stewart, Feng‐Juan Yang, Gong Cheng, Xiao‐Di Wang, Ding‐Ying Wang, Song‐Guang Xie

**Affiliations:** ^1^ State Key Laboratory of Marine Resource Utilization in South China Sea Hainan University Haikou China; ^2^ College of Ecology and Environment Hainan University Haikou China; ^3^ Department of Applied Ecology, Faculty of Environmental Sciences Czech University of Life Sciences Prague Prague Czech Republic; ^4^ Cawthron Institute Nelson New Zealand; ^5^ China Water Resources Pearl River Planning Surveying & Designing Co., Ltd. Guangzhou China; ^6^ Shenzhen Academy of Environmental Sciences Shenzhen China

**Keywords:** carbon source, diet assimilation, food web, *MixSIAR*, periphyton, submerged hydrophyte

## Abstract

The utilization of food resources by aquatic consumers reflects the structure and functioning of river food webs. In lotic water systems, where food availability and predator–prey relationships vary with gradient changes in physical conditions, understanding diet assimilation by local communities is important for ecosystem conservation. In the subtropical Liuxi River, southern China, the relative contribution of basal resources to the diet assimilation of functional feeding groups (FFGs) was determined by stable carbon (^13^C) and nitrogen (^15^N) isotope analyses. The output of Bayesian mixing models showed that diatom‐dominated periphyton (epilithic biofilm), aquatic C_3_ plants (submerged hydrophytes), and suspended particulate organic matter (SPOM) associated with terrestrial C_3_ plants contributed the most to the diet assimilation of FFGs in the upper, middle, and lower reaches, respectively. The relative contribution of consumer diet assimilation was weighted by the biomass (wet weight, g/m^2^) of each FFG to reflect resource utilization at the assemblage level. From the upper to the lower reaches, the spatial variation in the diet assimilation of fish and invertebrate assemblages could be summarized as a longitudinal decrease in periphyton (from 57%–76% to <3%) and an increase in SPOM (from <7% to 51%–68%) with a notable midstream increase in aquatic C_3_ plants (23%–48%). These results indicate that instream consumers in the Liuxi River rely more on autochthonous production (e.g., periphyton and submerged hydrophytes) than on terrestrially derived allochthonous matter (e.g., terrestrial plants). The pattern of resource utilization by consumers in the mid‐upper Liuxi River is consistent with findings from other open subtropical and neotropical rivers and provides evidence for the riverine productivity model. Our study indicates that protecting inherent producers in rivers (e.g., periphyton and submerged hydrophytes) and restoring their associated habitats (e.g., riffles with cobble substrate) are conducive to aquatic ecosystem management.

## Graphical abstract

The instream consumers in the subtropical Liuxi River rely more on autochthonous production (e.g., periphyton and submerged hydrophytes) than on terrestrially derived allochthonous matter (e.g., terrestrial plants). The pattern of resource utilization by consumers in the mid‐upper Liuxi River is consistent with findings from other open subtropical and neotropical rivers and provides an evidence of the riverine productivity model. Our study indicates that protecting inherent producers in rivers (e.g., periphyton and submerged hydrophytes) and restoring their associated habitats (e.g., riffles with cobble substrate) are conducive to aquatic ecosystem management.

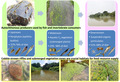

## 1 INTRODUCTION

To manage the integrity and functioning of river ecosystems, it is important to recognize the region‐specific characteristics of resource utilization by aquatic consumers (Wang et al., 2021; Welcomme et al., 2006; Zeni & Casatti, 2014). Several different theories have been developed to explain resource utilization in large river food webs and to provide a theoretical basis for the ecological functioning of large rivers, such as the river continuum concept (Vannote et al., 1980), the flood pulse concept (Junk, 1999), the riverine productivity model (Thorp & Delong, 1994), and riverine ecosystem synthesis (Thorp et al., 2006). Each of these models focuses on a different dimension of the riverscape. The river continuum concept has a longitudinal perspective and describes ecosystem processes from the upstream to downstream reaches, while the flood pulse concept highlights the importance of energy transfer from lateral floodplains.

In contrast, the riverine productivity model suggests that carbon sources supporting food webs in large river systems derive from within the river channel itself. The riverine ecosystem synthesis emphasizes that although these resources are predominantly autochthonous in nature, they can be influenced by the ecological nature of river hydrogeomorphic units. Therefore, resource subsidies to river food webs should have local particularity and be explored over a range of climates, hydrologic regimes, watershed geochemistry conditions, and anthropogenic pressures. Ecologists examined the origin of potential resources that fuel riverine ecosystem processes, and the results emphasized the relative contribution of allochthonous versus autochthonous organic matter to consumers (Pingram et al., 2012a). For example, in forested headwater streams, allochthonous inputs such as leaves may form the base of the food web; however, in wider rivers with low elevations, autochthonous algae may be more important to consumers. Beyond the acknowledged longitudinal pattern in increased reliance on algae in lotic systems, tropical headwater streams have been shown to have greater reliance on algal resources than their temperate counterparts (Lewis et al., 2001; March & Pringle, 2003). Collectively, these studies demonstrated specific resource utilization in different habitats, regions, and climate zones.

Studies examining the spatial pattern of resource utilization in temperate streams, characterized by fishes and insects, support different concepts (Hoeinghaus et al., 2007). However, previous studies based on behavioral–morphologic feeding habits and stomach/gut content analysis (Aarts & Nienhuis, 2003) leave questions regarding which and how resources are assimilated by consumers and their relative contribution to consumers' diet composition unanswered (Buchheister & Latour, 2015). Particularly, along a longitudinal gradient, the changing physical structure of a river determines the variable distribution of resources; thus, it is hypothesized that covariation exists between resource utilization by consumers and environmental changes across space (Chang et al., 2012; Hoeinghaus et al., 2007). As riverine organisms display different food sources across habitats, especially migratory assemblages and generalist feeders (e.g., omnivores), it is challenging to precisely quantify resource utilization by region‐ or site‐specific consumers.

Stable isotopes can be used to identify basal food resources used by consumers and determine broad‐scale linkages between consumers and organic matter sources. Terrestrial and aquatic plants often differ in their ^13^C signatures (Fry, 1991). Because the ^13^C signatures of primary consumers reflect those of the plants they eat, analyses of ^13^C signatures can be used to estimate the relative contributions of different basal resources in stream food webs (Finlay, 2001; March & Pringle, 2003; Rounick & Hicks, 1985). Analysis of ^13^C signatures provides an advantage over stomach/gut content analysis because the former measures the amount of carbon assimilated from each food source as opposed to the amount ingested. This differentiation is important because food sources can differ greatly in quality. Furthermore, stable isotopes are also useful in determining trophic levels because the ^15^N signature is generally enriched by 2.5–3.5‰ with every trophic transfer (Neres‐Lima et al., 2016; Peterson & Fry, 1987).

Due to spatially unbalanced development along disturbed river systems (e.g., upstream pristine forests, midstream agricultural areas, and downstream urbanized regions; Chang et al., 2012; Kaymak et al., 2018; Wang et al., 2018a), we hypothesized that the food resources used by local instream communities would be region‐ or site‐specific. We examined the δ^13^C and δ^15^N values of basal resources and aquatic consumers in heterogeneous habitats along a subtropical river in southern China. A Bayesian mixing model was used to analyze the relative contributions of basal resources, including periphyton, aquatic C_3_ plants (i.e., hydrophytes), terrestrial C_3_ and C_4_ plants (i.e., pasture grasses), and suspended particulate organic matter (SPOM), to the diet assimilation of fish and aquatic invertebrates. Differentiation between terrestrial C_3_ and C_4_ plants often indicates a catchment land‐use transition from low intensity/fellow land cover (C_3_) to high intensity/improved agriculture (C_4_).

We aimed to test the hypothesis by addressing the following questions: (1) What are the relative contributions of various resources to aquatic consumers and assemblages? (2) Do the contributions of these resources vary along rivers whose natural continuum is disrupted by human activities? (3) Which concept best explains the spatial pattern of resource utilization along the studied subtropical river? By answering these questions, we can gain insight for evaluating the effects of ongoing management activities and potential restoration measures on stream or river ecosystems.

## 2 MATERIALS AND METHODS

### 2.1 Study area and sampling sites

**FIGURE 1:**
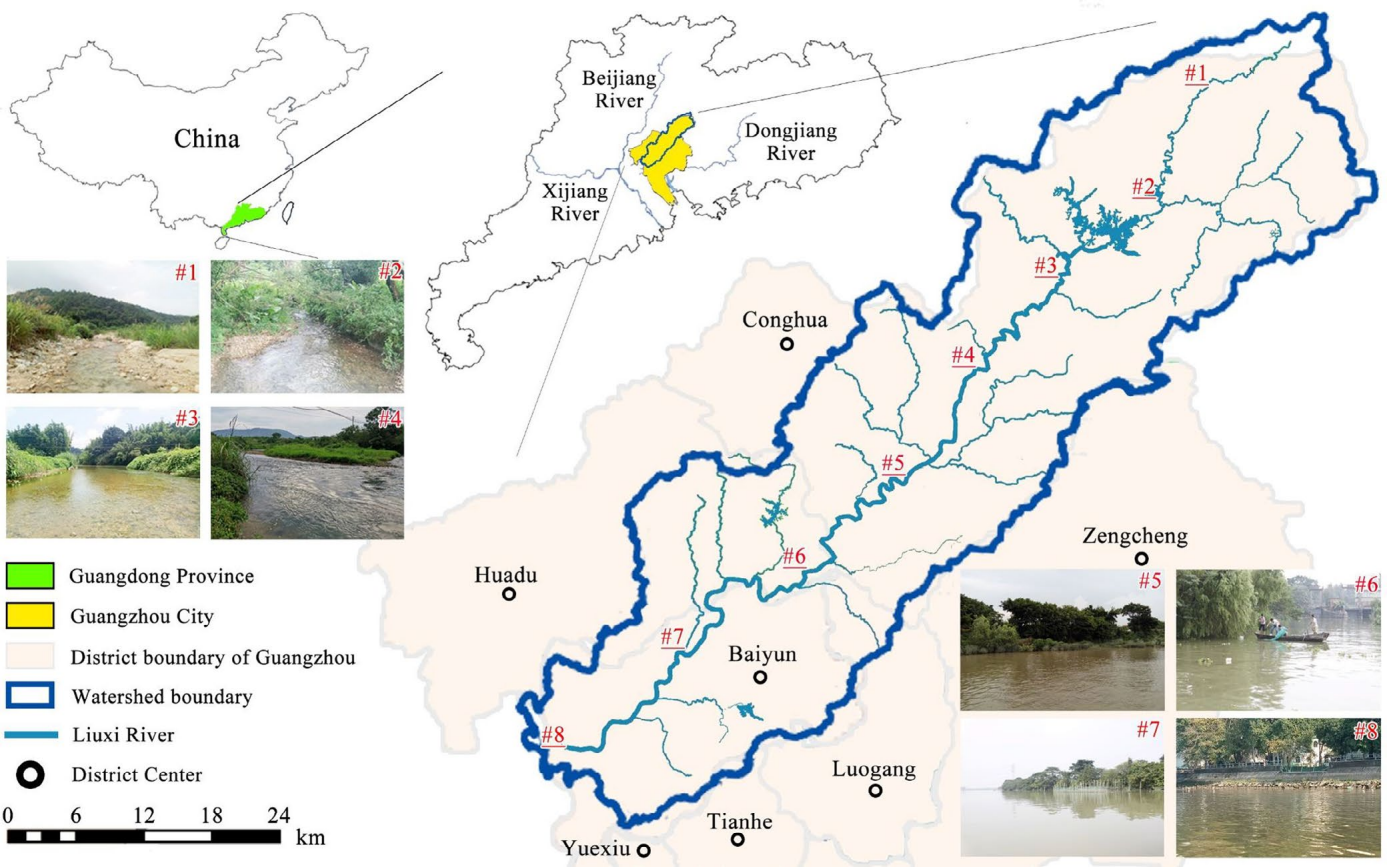
Locations of the eight sampling sites (#1–#8) along the main channel of the Liuxi River

The Liuxi River is regarded as the mother river (of high cultural significance) of Guangzhou, the capital city of Guangdong Province in southern China. The study area has a typical subtropical monsoon climate. The mean annual precipitation of the watershed is 1800 mm, mostly occurring in April–September. The Liuxi River watershed (with an area of 2300 km^2^) is situated in the northeastern corner of the Pearl River Delta, which has experienced rapid development in the last two decades. The watershed spans four county‐level districts (Huadu, Luogang, Baiyun, and Conghua), which occupy 70% of the watershed area (Figure 1). The water of the Liuxi River is used for a wide range of purposes, such as drinking water supply, agriculture and industry water use, and recreation. However, the source‐water intake near the river outlet (site 8) was abandoned in 2010 due to pollution, and only intakes located in the middle river (sites 5–6) are still in use.

Eight sampling sites, extending from headwaters to the estuary, were chosen along the main channel of the Liuxi River (Figure 1). Environmental data, including habitat characteristics and physicochemical parameters of water quality (see Tables S1 and S2 of the Supporting Information), were provided by a nationally accredited (China Metrology Accreditation) third‐party testing agency. Forests are the dominant land use type in the watershed, accounting for 55% of the total area. Approximately 33% of the watershed is used for agriculture, including orchards (20%), paddy rice fields (9%), and vegetable lands (4%). The rest of the watershed is occupied by built‐up areas (9%) and water (3%). The development in the watershed is spatially unbalanced. The upstream areas (sites 1–2) are covered by dense forests, while agricultural activities are concentrated in the midstream areas (sites 3–5). The area close to the watershed outlet (sites 6–8) is highly urbanized.

### 2.2 Sample collection and functional feeding group classification

Consumer and basal resource samples were collected concurrently from headwater site 1 to downstream site 8 in September 2020, which is the transition period between the rainy (June to July) and dry (November to December) seasons. The methods for collecting primary producers (e.g., periphyton, aquatic C_3_ plants, and terrestrial C_3_ and C_4_ plants), aquatic consumers (fish, shrimp, crabs, insects, bivalves, gastropods, annelids, and zooplankton), and suspended particulate organic matter (SPOM) are provided in the supplementary methods of the Supporting Information. Macroinvertebrate and fish specimens were sampled and quantified in wet weight (g/m^2^) according to the methods for building mass‐balance Ecopath models by Wang et al. (2018a). All consumer species were identified to the lowest possible taxonomic level and assigned to FFGs following the classification of Morse et al. (1994) and Wang et al. (2019). Zooplankton specimens included cyclopoid copepods and malacostracan nauplii that could be selected while alive under stereomicroscopy. The dominant species/genera/families and FFGs of fish and invertebrates are provided in Table S3 of the Supporting Information.

### 2.3 Preparation and determination for stable isotope analysis

According to previous basin‐scale faunal surveys along the Liuxi River (data provided by Guangzhou Environmental Monitoring Center Station), the fish and invertebrate species in each FFG can represent the dominant populations of the local aquatic community (Wang et al., 2020c). Aquatic insects, tadpoles, snails, amphipods, and polychaetes were kept for 1 day in clean water to allow for gut evacuation. For small insect larvae, zooplankton, and amphipods, multiple individuals from the same taxon were pooled as replicates, and the entire composite was used. For large shrimp, crabs, snails, odonates, and fish, individual specimens were treated as replicates, and only the muscle tissues were used. All samples were freeze‐dried, ground into homogeneous powder, and filtered through a 150‐mesh sieve (pore size 100 µm). The sample size for stable isotope measurement of each FFG at each site is provided in Table S3 of the Supporting Information.

Samples of SPOM, zooplankton, bivalves, gastropods, and crustaceans were tested for the presence of carbonates by adding a few drops of 10% HCl to each subsample. Given that acidification can affect δ^15^N signals, a nonacidified subsample was always kept for separate nitrogen isotope analysis (Carabel et al., 2006). C:N ratios were used to normalize δ^13^C for lipid content. For the fish and invertebrate samples with C:N values >3.5, such as bivalves (C:N = 4.45–5.78), insect predators (C:N = 4.17–5.31), and shrimp scrapers (C:N = 4.30–5.25), the δ^13^C values were normalized according to the equation by Post et al. (2007):

$$\delta^{13}C_{\text{normalized}}=\delta^{13}C_{\text{untreated}}‐3.32+0.09\times C:N$$

where δ^13^C_normalized_ is the normalized estimate of δ^13^C for the effects of lipid concentration and δ^13^C_untreated_ is the δ^13^C of samples determined without lipid extraction.

Stable isotope data were reported as the difference between the ratios of a sample and standard, using the standard notation of δ^13^C or δ^15^N (‰) = [(X_sample_/X_standard_) − 1] × 10^3^, where X = ^13^C/^12^C or ^15^N/^14^N. δ^13^C or δ^15^N is the per‐mil (‰) deviation of that sample from the recognized isotope standard: Vienna Pee Dee Belemnite for δ^13^C and atmospheric N_2_ for δ^15^N. The δ^13^C and δ^15^N values of the samples were determined with a continuous‐flow isotope ratio mass spectrometer (Finnigan Delta S) coupled with an elemental analyzer (NA 1500; Fison, Milan, Italy) in the Stable Isotope Laboratory, Third Institute of Oceanography, China. The precision of repeated measurements of the standard reference materials was ±0.1‰ for both the carbon and nitrogen stable isotopes. The standard error of the mean for replicates of the same tissue was 0.13‰ for δ^13^C and 0.15‰ for δ^15^N.

### 2.4 Statistical analysis

A Bayesian mixing model (R package *MixSIAR*, R Core Team; Parnell et al., 2010) was used to estimate the relative contribution (%) of basal resources to the diet assimilation of invertebrate and fish FFGs based on stable isotope values. Prior to analysis, the δ^13^C‐δ^15^N space of consumer FFGs and five basal resources at sampling sites 1–8 were plotted to diagnose the potential solutions (see the convex hull in Figure S1 of the Supporting Information). The δ^13^C and δ^15^N values of each consumer specimen were input as raw data, whereas those of basal resources were input as the means and standard deviations. Although the stable isotope data were correlated, *MixSIAR* assumes multivariate normality, which accounts for the fact that tracer values can covary. The model is fit via Markov chain Monte Carlo, which estimates entire posterior distributions for each variable. Aquatic C_3_ plants were not used as a source in the *MixSIAR* model at site 1 because submerged hydrophytes did not appear in the headwaters. To back‐calculate the underlying sources, we used a δ^13^C trophic fractionation of 0.96 ± 0.26‰ for all consumers and δ^15^N trophic fractionation of 1.07 ± 0.32‰ for invertebrates and 2.38 ± 0.37‰ for fish (Wang et al., 2020a).

To reflect the overall trend of resource utilization by local assemblages, the relative contributions of basal resources to the diet composition of fish and aquatic invertebrate assemblages were calculated as:

$$RC_{m}=\frac{R_{m}\times B_{j}}{\sum_{j=1}^{n}R_{i}\times B_{j}}$$

where *RC_m_* is the relative contribution of resource *m* (e.g., periphyton) to the diet of a consumer assemblage (i.e., invertebrate or fish); *B_j_* is the biomass (fresh weight, g/m^2^) of functional feeding group *j* (*j *= 1 − *n*), where *n* is the highest number of local functional feeding groups; and *R_m_* is the relative contribution of resource *m* to the diet assimilation of functional feeding group *j*.

Nonmetric multidimensional scaling (NMDS) was used to visualize the relationships between consumers and their food resources based on the diet assimilation data matrix output by the *MixSIAR* model (i.e., relative contributions [%] of five resources to the diet assimilation of fish and invertebrate FFGs). NMDS is a nonparametric ordination technique that relies on the rank order of pairwise dissimilarities (Euclidean distance in this study) and does not make any underlying distributional assumptions about the data (Borcard et al., 2011). NMDS was chosen over parametric ordination approaches because the diet assimilations of consumer FFGs at sites 1–8 were skewed and not normally distributed. The sampling sites were plotted in the ordination space with the distance among points positively related to the dissimilarity of the consumer diet assimilation; thus, fish and invertebrate FFGs that used similar basal resources were plotted closer to one another.

A structural equation model (SEM), including both confirmatory factor analysis and path analysis in particular, was applied to explore the direct, indirect, and total effects of environmental variables on consumers' diet assimilation. As a multivariate statistical methodology, SEM involves sets of multiple regression analyses, where the causal relationships between variables can be examined with multiple linear equations. The proportion of the variance explained by each variable can be indicated by *R* ^2^ values. The standardized path coefficients, which were calculated by multiplying the unstandardized partial regression coefficients by the ratio of the standard deviations of the explanatory variables on either end of a path, represent the relative strengths of the direct effects within the SEM. The Chi‐square (χ^2^) test was used to assess the fit between the predicted and observed covariance matrices. A χ^2^ result with *p* > .05 indicates an acceptable model fit with the observed covariance structure. The fitted models were also assessed based on Bentler's comparative fit index (CFI) and root mean square error of approximation (RMSEA). A CFI > 0.90 and RMSEA < 0.10 were considered acceptable (Grace et al., 2010). The SEM was fitted using the R packages *lavaan* and *semPlot*. We evaluated the goodness of fit with Fisher's test after removing nonsignificant pathways between explanatory and latent variables from the SEM using the R package *piecewiseSEM*.

## 3 RESULTS

### 3.1 Relative contributions of basal resources to the diet assimilation of invertebrate FFGs

**FIGURE 2:**
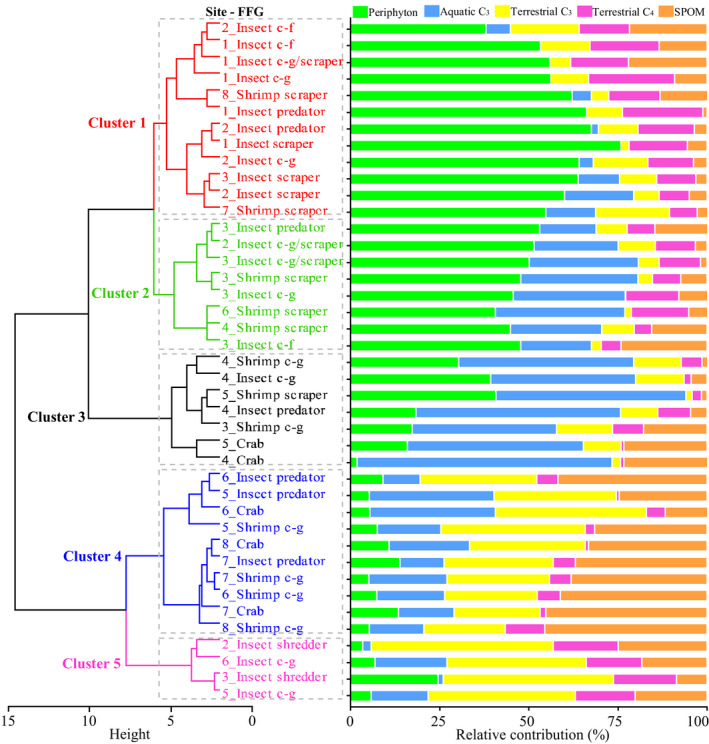
Cluster analysis based on the relative contributions (%) of five basal resources to the diet assimilation of invertebrate functional feeding groups (FFGs). SPOM, suspended particulate organic matter; c–g, collector–gatherer; c–f, collector–filterer

The diet assimilation of invertebrate FFGs showed two spatial patterns (Figure 2), one along the longitudinal gradient (i.e., from the upper to the lower reaches) and the other across the lateral gradient (i.e., from the riparian or revetment zones to the central channel). The shrimp scrapers (Atyidae: *Neocaridina denticulata*), which live near the littoral zone that has lush water grass, showed less longitudinal variation in diet assimilation. Thus, even if shrimp scrapers were sampled at downstream sites 7–8, they were grouped with the insect groups (e.g., insect c‐g, c‐f, scraper, and c‐g/scraper) that were typically distributed at upstream sites 1–2. Except for those of shrimp scrapers, all the invertebrate FFGs showed obvious longitudinal variation in diet assimilation, which could be generally divided into the upper (sites 1–2), mid‐upper (sites 3–4), mid‐lower (sites 5–6), and lower (sites 7–8) reaches. The basal principles identified in these reaches were the great contribution (%) of periphyton to the diet assimilation of Cluster 1, the increased contribution of aquatic C_3_ plants to the diet assimilation of Cluster 2, the greatest contribution of aquatic C_3_ plants to the diet assimilation of Cluster 3, and the increased contribution of SPOM and terrestrial C_3_ plants to the diet assimilation of Cluster 4. Cluster 5 was an exception because it was composed of insect shredders that preferred to eat terrestrial C_3_ and C_4_ plant debris. In addition, insect c‐gs at sites 5–6 could be sampled only in the littoral zone due to the high water depth, and decaying terrestrial plant debris might provide the dominant food sources for the local insect c‐gs.

### 3.2 Relative contributions of basal resources to the diet assimilation of fish FFGs

**FIGURE 3:**
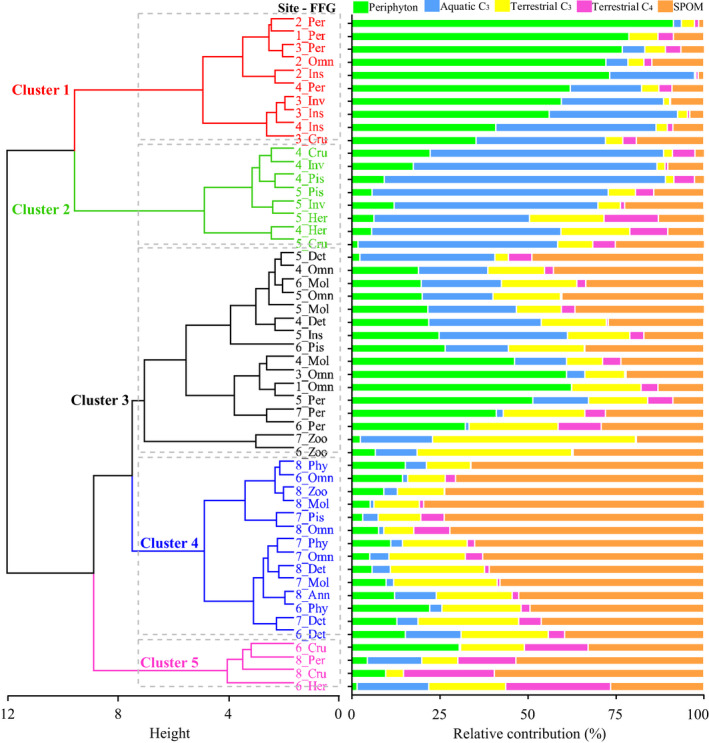
Cluster analysis based on the relative contributions (%) of five basal resources to the diet assimilation of fish functional feeding groups (FFGs). SPOM, suspended particulate organic matter. Per, periphytivore; Ins, insectivore; Omn, omnivore; Det, detritivore; Inv, invertivore; Mol, molluscivore; Cru, crustaceavore; Pis, piscivore; Phy, phytoplanktivore; Zoo, zooplanktivore; Her, herbivore; Ann, annelidivore

Similar to the pattern observed for invertebrate FFGs, fish FFGs were also divided into five clusters according to the relative contributions of five basal resources in the diet assimilation (Figure 3). Clusters 1, 2, and 4 were identified by the greatest contribution (in terms of the mean) of periphyton, aquatic C_3_ plants, and SPOM to diet assimilation, respectively; Cluster 3 was identified by the mixed contribution of periphyton and aquatic C_3_ plants to diet assimilation; and Cluster 5 was identified by the high contribution of terrestrial C_4_ plants to diet assimilation. Compared with that of invertebrates, the longitudinal variation in resource utilization of fish showed a more obvious changing trend from periphyton to submerged hydrophytes and to SPOM along the river, suggesting that the diet assimilation of fish might reflect the long‐term status of the local resource supply.

Resource utilization by fish assemblages showed significant differences among FFGs and exhibited three spatial patterns from the headwaters to the lower reaches: decreased periphyton with increased SPOM in the diet assimilation of periphytivorous, omnivorous, and molluscivorous fish; decreased periphyton with increased aquatic C_3_ plants in the diet assimilation of insectivorous and invertivorous fish; and decreased aquatic and terrestrial C_3_ plants with increased SPOM in the diet assimilation of crustaceavorous, piscivorous, detritivorous, phytoplanktivorous, and zooplanktivorous fish. These results indicated the dependence of fish FFGs on local food resources. Considering the longitudinal shift in the resources assimilated by invertebrate FFGs, in addition to local basal resources, the diet assimilation of fish may also be influenced by their consumption of invertebrates with different isotope signatures.

### 3.3 Comparison of diet assimilation among the site‐specific consumer assemblages

**FIGURE 4:**
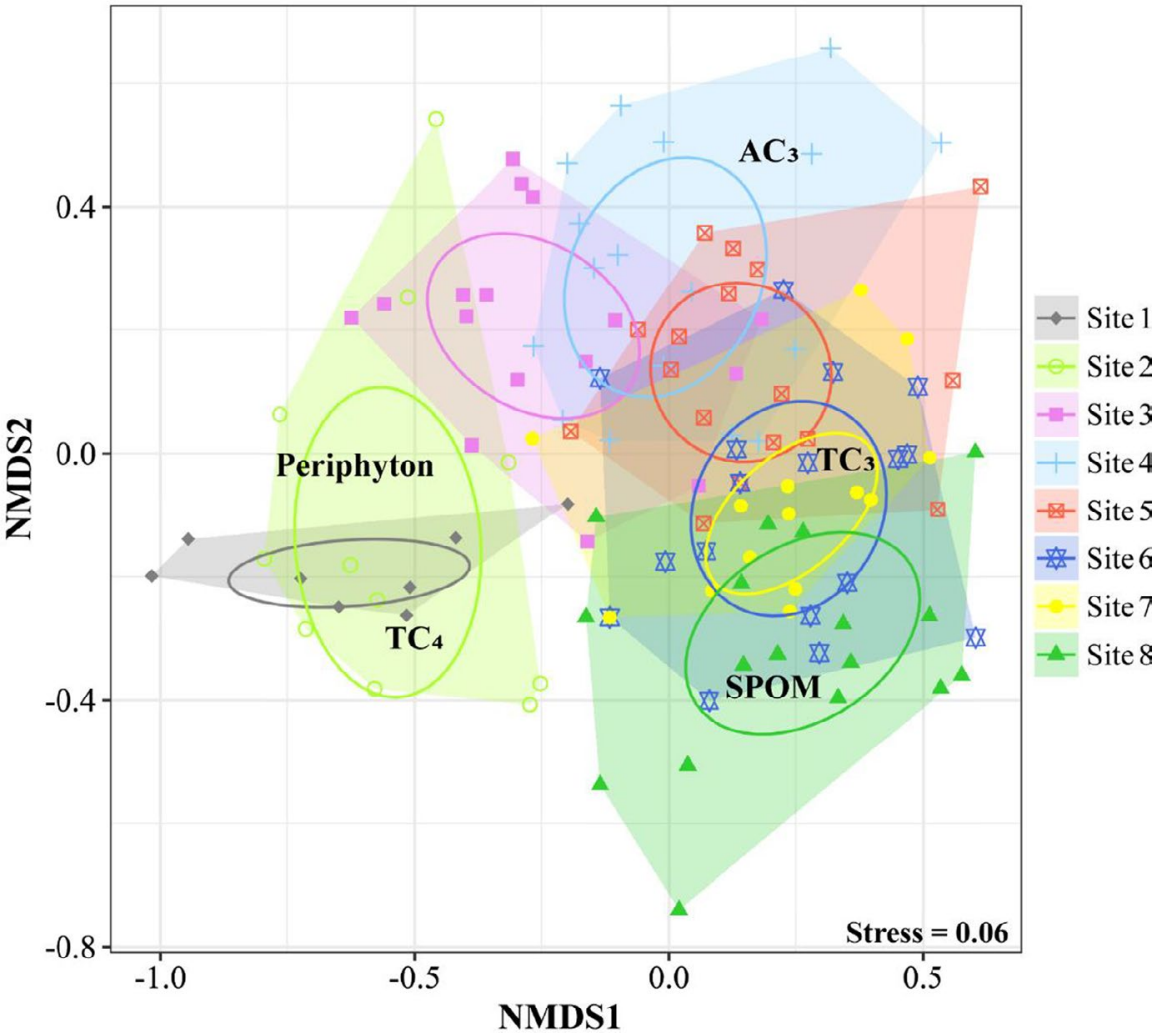
Nonmetric multidimensional scaling (NMDS) based on the relative contributions (%) of five resources to the diet assimilation of site‐specific consumer assemblages composed of fish and invertebrate functional feeding groups. AC_3_, aquatic C_3_ plants; TC_3_, terrestrial C_3_ plants; TC_4_, terrestrial C_4_ plants; SPOM, suspended particulate organic matter

The NMDS ordination ranked consumer assemblages composed of fish and invertebrate FFGs based on the relative contributions (%) of five resources to diet assimilation (Figure 4). The NMDS stress value of 0.06 shows a good fit. From site 1 to site 8, there was a longitudinal shift in dominant resource utilization by site‐specific consumer assemblages, which was reflected by the clockwise distribution of five resources from the 3rd to the 2nd quadrant in the NMDS biplot. In the upper stream (sites 1–2), periphyton, located near the center left of the biplot composed of the NMDS1 and NMDS2 axes, was an important resource used by local consumers. In the mid‐upper reaches (sites 3–5), aquatic C_3_ plants could be regarded as an important resource because they were close to the center of the 1st quadrant in the NMDS biplot. In the mid‐lower reaches (sites 6–8), terrestrial C_3_ plants, located near the center right of the NMDS biplot, were dominant resources used by local consumers. In addition to terrestrial C_3_ plants, SPOM was an important resource for the consumers at estuarial site 8. Notably, the increased overlap area of a convex hull and Bayesian ellipses between sampling sites indicated a high degree of commonality in resource utilization by the consumers in the lower reaches.

### 3.4 Biomass‐weighted resource utilization by invertebrate and fish assemblages

**FIGURE 5:**
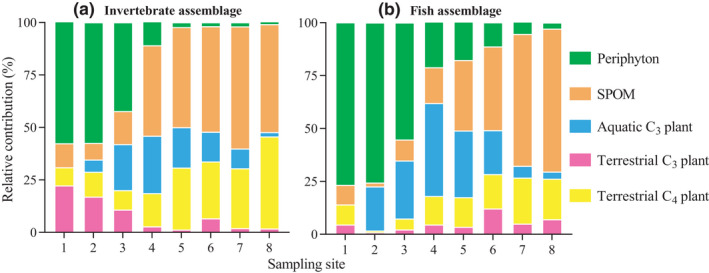
Relative contributions (%) of five basal resources to the diet assimilation of invertebrate and fish assemblages. S1–S8, sampling sites 1–8; SPOM, suspended particulate organic matter

At each site, the relative contributions (%) of the five resources to the diet assimilation were weighted by the biomass (wet weight, g/m^2^) of invertebrates (Figure 5a) and fish (Figure 5b) to reflect the overall trend of resource utilization by local assemblages (unpublished original biomass data). The biomass‐weighted diet assimilation eliminated the interference of the diet assimilation of low‐biomass FFGs on the diet assimilation of the whole assemblage. Generally, the spatial variation in consumer diet assimilation could be summarized as a longitudinal decrease in the contribution of periphyton with an increase in the contribution of SPOM. This pattern demonstrated assemblage‐level diet assimilation that could not be reflected by the diet assimilation of individual FFGs. Notably, in the middle reaches (sites 3–6), submerged aquatic C_3_ plants played an important role in determining the contribution of autochthonous organic matter to resource utilization by local consumers. For example, at sites 4–5, autochthonous organic matter contributed >50% of the resource supply in the diet assimilation of fish and invertebrates, with aquatic C_3_ plants contributing 20%–30%. The contribution of aquatic C_3_ plants, to some extent, might determine the main type of organic matter supply (i.e., autochthonous vs. allochthonous) used by local aquatic communities.

### 3.5 Evaluating the effects of environmental variables on system attributes

**FIGURE 6:**
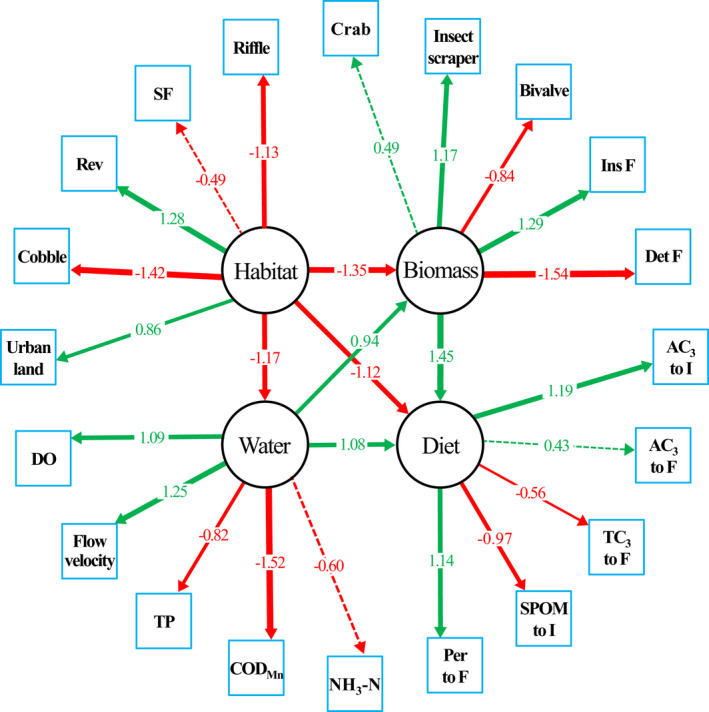
Structural equation models (SEMs) showing the direct and indirect effects of environmental factors, including habitat characteristics, water quality, and biomass composition, on the diet assimilation of fish and invertebrate assemblages. Green and red arrows indicate positive and negative effects, respectively. Solid and dashed lines indicate significant (*p* < .05) and nonsignificant (*p* > .05) relationships, respectively. The width of the arrows is proportional to the strength of the path coefficients (numbers adjacent to arrows). The numeric values are the standardized path coefficients and variance estimates. Riffle habitat, % of habitat area; SF, seasonal floodplain area (m^2^); Rev, revetment (% of riverbank length); cobble substrate, % of substrate area; urban land, % of land use area. DO, dissolved oxygen (mg/L); flow velocity (m/s); TP, total phosphorus (mg/L); COD_Mn_, chemical oxygen demand determined using the permanganate method (mg/L); NH_3_‐N, ammonia nitrogen (mg/L). Crab (g/m^2^); insect scraper (g/m^2^); bivalve (g/m^2^); Ins F, insectivorous fish (g/m^2^); Det F, detritivorous fish (g/m^2^). AC_3_ and SPOM to I, relative contribution (%) of aquatic C_3_ plants and suspended particulate organic matter to the diet assimilation of invertebrate assemblages. AC_3_, TC_3_, and Per to F, relative contribution (%) of aquatic C_3_ plants, terrestrial C_3_ plants, and periphyton to the diet assimilation of fish assemblages

The accepted SEM explained a moderate amount of the variance in richness (60%) and fit the data well, with an RMSEA of 0.061 (*p* = .056) and a goodness‐of‐fit index (GFI) value of 0.94 (Figure 6). These index values showed that there was a strong correlation between the predicted and observed covariances. The direct paths from riffle habitat, seasonal floodplain, riparian revetment, cobble substrate, and urban land to the latent variable ‘habitat’ were −1.13, −0.49, 1.28, −1.42, and 0.86, respectively, referring to the shift from upstream pristine habitats to downstream disturbed habitats. The direct paths from dissolved oxygen (DO), flow velocity, total phosphorus (TP), chemical oxygen demand (COD_Mn_), and ammonia nitrogen (NH_3_‐N) to the latent variable ‘water’ were 1.09, 1.25, −0.82, −1.52, and −0.60, respectively, indicating longitudinal deterioration in water quality (e.g., decreased DO and increased nutrients). The direct paths from crabs, insect scrapers, bivalves, insectivorous fish, and detritivorous fish to the latent variable ‘biomass’ were 0.49, 1.17, −0.84, 1.29, and −1.54, respectively, indicating a longitudinal shift in biomass composition from rheophilic to limnophilic assemblages. The direct paths from ‘habitat’ to ‘water’, ‘biomass’, and ‘diet’ were −1.17, −1.35, and −1.12, respectively, indicating that the degraded habitat conditions from upstream to downstream had strong negative impacts on other variables. In contrast, the direct paths from ‘water’ to ‘biomass’ and ‘diet’ were 0.94 and 1.08, respectively, indicating that excellent water quality with high DO and flow velocity had positive impacts on both biomass and diet composition.

## 4 DISCUSSION

### 4.1 Region‐specific resource utilization in the upper stream

Terrestrial plants are rarely limited by the CO_2_ supply, and thus have δ^13^C fractionation consistent with that of atmospheric CO_2_, resulting in relatively constant δ^13^C values of −28.0 ± 0.9‰ for C_3_ plants and −13.5 ± 1.5‰ for C_4_ plants (Finlay, 2001). This result contrasts with the periphyton δ^13^C values of −18.5 ± 2.7‰ (commonly −30‰ to −15‰), which vary as a function of productivity and flow velocity (Finlay, 2004). In addition, periphyton δ^13^C values might also be influenced by periphyton composition, that is, seasonally dependent taxonomic composition of bacteria and algae, which can store large amounts of carbon and nutrients (Wu, 2016). Aquatic (e.g., periphyton) and terrestrial vegetation (e.g., vascular C_3_ plants) form the diets of invertebrates or fish in streams with different climates, producing local coupling between consumers and local resources (Jardine & Bunn, 2012; March & Pringle, 2003; Pingram et al., 2012a). Diatom‐dominated periphyton (epilithic biofilm) appeared to be the main autochthonous carbon source driving secondary production in the upper wadable stream (sites 1–3) of this subtropical urban river in the Northern Hemisphere. Previous investigations in tropical systems also reported that periphyton, despite forming an inconspicuous component of the total carbon biomass, is largely responsible for metazoan biomass (Bunn et al., 2003; Thorp & Delong, 2002; Wang et al., 2018b), whereas detritus from terrestrial vascular C_3_ and C_4_ plants plays only a minor role (Lau et al., 2009; Lewis et al., 2001).

Our finding is consistent with those of studies on large rivers in Australia (Bunn et al., 2003; Medeiros & Arthington, 2011), the subtropics (e.g., the East River in southern China, Wang et al., 2020b), and the neotropics (e.g., the Parana´ and Orinoco Rivers in South America, Hoeinghaus et al., 2007; Jepsen & Winemiller, 2007), where benthic algae provide important carbon sources for food webs in lower order streams. In contrast, some large continental rivers (e.g., the Mississippi and Ohio Rivers in North America, Delong & Thorp, 2006; Herwig et al., 2004; Thorp et al., 1998) in temperate and dry climates rely more on the terrestrial carbon supply that provides inputs as coarse organic particulates. In temperate streams, the low temperature, seasonal dryness, and dense canopy limit autochthonous production; thus, most primary consumers use allochthonous matter (e.g., riparian leaf litter, see Chang et al., 2012; Kaymak et al., 2018). In the tropics and subtropics, however, constant sunlight, an open canopy, perennial precipitation, and a wet climate stimulated the productivity of epilithic diatoms (e.g., *Melosira* and *Aulacoseira*, Wang et al., 2018a). Notably, the canopy immediately above the headwater streams was not covered by dense forests, because the riparian vegetation was composed of straight‐growing eucalyptus and bamboo. As a result, diatom‐dominated periphyton was the major carbon source for consumers in the upper Liuxi River, which explained the high contribution of periphyton to the diet assimilation of consumer FFGs at sites 1–3.

### 4.2 Longitudinal shifts in resource utilization by consumer FFGs along the stream

**FIGURE 7:**
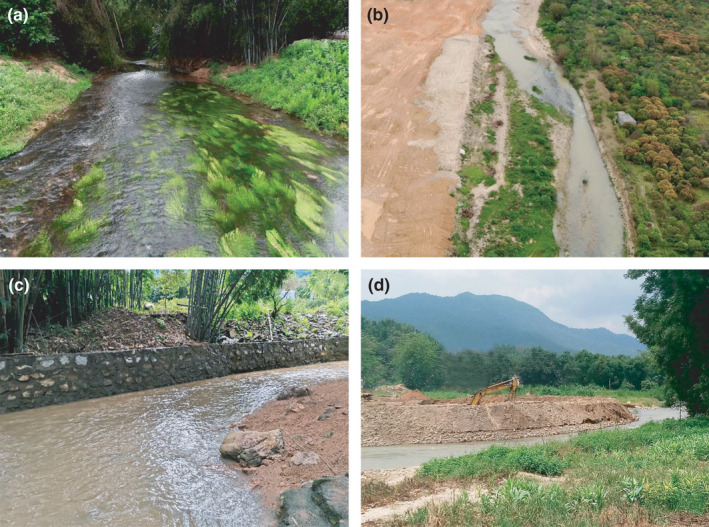
Undisturbed (a) and disturbed (b–d) habitats in the Liuxi River. (a) Submerged macrophyte zone at site 3. (b) Riverbed modified by land‐cover changes. (c) Revetments and channelization. (d) Gravel extraction at site 7

An interesting finding was that submerged hydrophytes (e.g., *H. verticillata* and *M*. *verticillatum*) played an important role as a resource supply in the transition zone connecting the upper wadable and middle nonwadable stream sections (Figure 7a), which enhanced the effects of autochthonous resources on the energy supply of the local food web. Similarly, Hoeinghaus et al. (2007) examined carbon flow in 10 food webs of the Paraná River, Brazil, and found that aquatic C_3_ plants and phytoplankton were the dominant (>75%) carbon sources in low‐gradient rivers and reservoirs, respectively. Moreover, the longitudinal pattern of resource utilization by consumer FFGs in the Liuxi River was almost the same as that reported in the Waikato River of New Zealand (Pingram et al., 2012b), where the morphological features of hydrophytes and feeding habitats of fish/invertebrates in the two regions were similar. By comparing the habitat features provided in these studies, we speculated that, regardless of climate zone, the open canopy cover, pristine riffle habitats and cobble/gravel substrates, as well as the undisturbed hydrologic conditions (e.g., shallow depth and high flow velocity in the upper stream), were the determinants influencing the region‐specific carbon flows (Figure 6, also see Wang et al., 2018b; 2020c). Given that aquatic vegetation serves as a key substrate for biofilms as well as a habitat and refugium for aquatic organisms, riverine invertebrates prefer to dwell and hide in submerged hydrophytes, and thus, they often scrape the periphyton attached to the blade surface (Wu, 2016). This may also explain why periphyton dominates the consumers' diet assimilation in the upper river. The availability and accessibility to such essential habitats, which support the high productivity of benthic periphyton and biofilms, might be critical to carbon flow transmission along periphyton‐based food chains.

From headwaters to the lower reaches, a downstream decrease in periphyton and aquatic C_3_ plants with an increase in SPOM and terrestrial C_3_ plants, which indicated the longitudinal replacement of autochthonous by allochthonous organic matter along the river, was the basic spatial pattern for the diet assimilation of widespread invertebrate and fish FFGs (Figures 4 and 5). In contrast to the features of the upstream riffle habitats, the increased water depth, decreased flow velocity, and replacement of cobble substrate by sand/silt in the middle‐lower reaches considerably lessened the production of both periphyton and associated consumer communities (e.g., insect FFGs and periphytivorous/insectivorous fish, Wang et al., 2018a). This difference might explain the shift in resource use from upstream periphyton to downstream SPOM and terrestrial C_3_ plants. In addition, the river corridors in rural and urban areas are facing intense anthropogenic pressures, particularly with respect to vegetation degradation (Figure 7b), revetments and channelization (Figure 7c), and gravel extraction (Figure 7d). These human disturbances have strong negative impacts on the properties and functioning of local ecosystems. From the perspective of primary energy supply, our results indicated the importance of protecting pristine habitats with high periphyton and hydrophyte productivity, such as riffles, cobble/pebble substrates, and floodplains (see Figure 6).

### 4.3 A suitable concept for explaining the pattern of resource utilization

Carbon flows in large river food webs are dependent on variations in habitat conditions, both temporally in terms of flow variability and spatially in relation to habitat characteristics. Based on recent work using isotope analysis to quantify carbon flows in large river food webs (Pingram et al., 2012a), it appears that autochthonous carbon of benthic algae and, to a lesser extent, hydrophytes and phytoplankton provide the dominant carbon sources fuelling the riverine food webs. This pattern was also observed within the present study for the diet assimilation of invertebrate assemblages at sites 1–3 and of fish assemblages at sites 1–5 (Figure 5), indicating that aspects of the riverine productivity model supported by recent literature were most appropriate for describing the carbon sources of food webs in the upper and middle Liuxi River. In contrast, a downstream increase in the contribution of terrestrial detritus of C_3_ and C_4_ plants along the river indicated the increased importance of allochthonous carbon for the food webs in the lower Liuxi River. Importantly, the Liuxi River is influenced by increasing basin‐scale anthropogenic pressures, especially dam interception and concrete revetment, and natural flow regimes and original habitats are modified, leading to a loss of riverscape connectivity (e.g., disappearing floodplains and riffles). Such serious discontinuity and fragmented habitats obstruct the input of terrestrial organic matter from the upper to the lower reaches and from riparian zones to open waters.

Given that SPOM was a mixed source of phytoplankton and suspended particulates, it was difficult to identify the contribution of phytoplankton and terrestrial plants. In a general observation under the microscope, more than 90% (volumetric percentage) of the SPOM was composed of plant detritus. Because there are few floodplains in the lower Liuxi River, particulate detritus in SPOM might be the decaying plant debris transported from the surrounding vegetation zones. In this case, the explanatory power of riverine productivity model for carbon utilization in the lower Liuxi River seems to be weakened due to the decreased contribution of primary producers in diet assimilation. However, the water level of the Liuxi River half a century ago was shallow enough that local villagers could walk across it, even at site 8 (interviews with local fishers >60 years of age). At that time, the distribution of riffle habitats with cobble/pebble substrates was extended to the lower reaches, where there were only sand/silt substrates. We speculate that, compared with the current river conditions, the water level would be much lower and the autochthonous productivity of periphyton would be higher without interception from dams. This indicates that riverine productivity model may explain the carbon utilization patterns along the Liuxi River without or with less human disturbance to pristine habitats.

## 5 CONCLUSIONS

This study assessed the spatial pattern of resource utilization by aquatic consumers along a subtropical river. Autochthonous resources, including cobble‐attached periphyton and aquatic C_3_ plants (e.g., submerged hydrophytes), were the dominant resources used by fish and invertebrates in the upper–middle reaches of the Liuxi River. In the middle–lower reaches, SPOM, consisting of phytoplankton and vascular plant detritus and, to a lesser extent, terrestrial C_3_ plants, provides the dominant carbon sources fuelling this river food web. Notably, aquatic C_3_ plants, which have received less attention in other research, are important for consumers in transition zones, that is, from upstream wadable to midstream nonwadable streams. Our results demonstrated that aspects of the riverine productivity model are most suitable for revealing the longitudinal pattern of resource utilization along this large, disturbed river system, which is more similar to other examples in the neotropics and tropics than to those in temperate areas. The findings in the Liuxi River have important implications for revealing energy supply and transmission processes in river food webs, which may have pronounced effects on the functioning of these aquatic ecosystems.

## CONFLICT OF INTEREST

The authors have no conflicts of interest to declare.

## AUTHOR CONTRIBUTIONS

**Sai Wang**: Conceptualization (lead); data curation (lead); formal analysis (lead); investigation (lead); methodology (lead); validation (lead); writing‐original draft (lead); writing‐review and editing (lead). **Tuan‐Tuan Wang**: Conceptualization (equal); data curation (equal); funding acquisition (equal); formal analysis (equal); investigation (equal); methodology (equal); writing‐review and editing (equal). **Wen‐Tong Xia**: Conceptualization (equal); data curation (equal); formal analysis (equal); investigation (equal); writing‐original draft (equal). **Zhong‐Bing Chen**: Conceptualization (equal); data curation (equal); project administration (equal); writing‐original draft (equal). **Simon D. Stewart**: Conceptualization (equal); data curation (equal); investigation (equal); writing‐review and editing (equal). **Feng‐Juan Yang**: Data curation (supporting); formal analysis (supporting); investigation (supporting); methodology (supporting). **Gong Cheng**: Data curation (supporting); formal analysis (supporting); investigation (supporting); methodology (supporting). **Xiao‐Di Wang**: Data curation (supporting); formal analysis (supporting); investigation (supporting). **Ding‐Ying Wang**: Data curation (supporting); formal analysis (supporting); investigation (supporting). **Song‐Guang Xie**: Funding acquisition (lead); project administration (equal); resources (lead); supervision (lead); writing‐review and editing (equal).

## Supporting information

Supplementary Material

## Data Availability

All data supporting this study are provided as supporting information accompanying this manuscript.

## References

[ece38304-bib-0001] Aarts, B. G. W. , & Nienhuis, P. H. (2003). Fish zonations and guilds as the basis for assessment of ecological integrity of large rivers. Hydrobiologia, 500, 157–178. 10.1023/A:1024638726162

[ece38304-bib-0002] Borcard, D. , Gillet, F. , & Legendre, P. (2011). Numerical ecology with R. Springer.

[ece38304-bib-0003] Buchheister, A. , & Latour, R. J. (2015). Diets and trophic‐guild structure of a diverse fish assemblage in Chesapeake Bay, U.S.A. Journal of Fish Biology, 86, 967–992. 10.1111/jfb.12621 25627041

[ece38304-bib-0004] Bunn, S. E. , Davies, P. M. , & Winning, M. (2003). Sources of organic carbon supporting the food web of an arid zone floodplain river. Freshwater Biology, 48, 619–635. 10.1046/j.1365-2427.2003.01031.x

[ece38304-bib-0005] Carabel, S. , Godínez‐Domínguez, E. , Verísimo, P. , Fernández, L. , & Freire, J. (2006). An assessment of sample processing methods for stable isotope analyses of marine food webs. Journal of Experimental Marine Biology and Ecology, 336, 254–261. 10.1016/j.jembe.2006.06.001

[ece38304-bib-0006] Chang, H.‐Y. , Wu, S.‐H. , Shao, K.‐T. , Kao, W.‐Y. , Maa, C.‐J. , Jan, R.‐Q. , Liu, L.‐L. , Tzeng, C.‐S. , Hwang, J.‐S. , Hsieh, H.‐L. , Kao, S.‐J. , Chen, Y.‐K. , & Lin, H.‐J. (2012). Longitudinal variation in food sources and their use by aquatic fauna along a subtropical river in Taiwan. Freshwater Biology, 57, 1839–1853. 10.1111/j.1365-2427.2012.02843.x

[ece38304-bib-0007] Delong, M. D. , & Thorp, J. H. (2006). Significance of instream autotrophs in trophic dynamics of the Upper Mississippi River. Oecologia, 147, 76–85. 10.1007/s00442-005-0241-y 16170563

[ece38304-bib-0008] Finlay, J. C. (2001). Stable‐carbon‐isotope ratios of river biota: Implications for energy flow in lotic food webs. Ecology, 82, 1052–1064. https://doi.org/10.1890/0012‐9658(2001)082[1052:SCIROR]2.0.CO;2

[ece38304-bib-0009] Finlay, J. C. (2004). Patterns and controls of lotic algal stable carbon isotope ratios. Limnology and Oceanography, 49, 850–861. 10.4319/lo.2004.49.3.0850

[ece38304-bib-0010] Fry, B. (1991). Stable isotope diagrams of freshwater food webs. Ecology, 72, 2293–2297. 10.2307/1941580

[ece38304-bib-0011] Grace, J. B. , Anderson, T. M. , Olff, H. , & Scheiner, S. M. (2010). On the specification of structural equation models for ecological systems. Ecological Monographs, 80, 67–87. 10.1890/09-0464.1

[ece38304-bib-0013] Herwig, B. R. , Soluk, D. A. , Dettmers, J. M. , & Wahl, D. H. (2004). Trophic structure and energy flow in backwater lakes of two large floodplain rivers assessed using stable isotopes. Canadian Journal of Fisheries and Aquatic Sciences, 61, 12–22. 10.1139/f03-139

[ece38304-bib-0014] Hoeinghaus, D. J. , Winemiller, K. O. , & Agostinho, A. A. (2007). Landscape‐scale hydrologic characteristics differentiate patterns of carbon flow in large‐river food webs. Ecosystems, 10, 1019–1033. 10.1007/s10021-007-9075-2

[ece38304-bib-0015] Jardine, T. D. , Pettit, N. E. , Warfe, D. M. , Pusey, B. J. , Ward, D. P. , Douglas, M. M. , Davies, P. M. , & Bunn, S. E. (2012). Consumer‐resource coupling in wet‐dry tropical rivers. Journal of Animal Ecology, 81, 310–322. 10.1111/j.1365-2656.2011.01925.x 22103689

[ece38304-bib-0016] Jepsen, D. B. , & Winemiller, K. O. (2007). Basin geochemistry and isotopic ratios of fishes and basal production sources in four neotropical rivers. Ecology of Freshwater Fish, 16, 267–281. 10.1111/j.1600-0633.2006.00218.x

[ece38304-bib-0017] Junk, W. (1999). The flood pulse concept of large rivers: Learning from the tropics. Large Rivers, 11, 261–280. 10.1127/lr/11/1999/261

[ece38304-bib-0018] Kaymak, N. , Winemiller, K. , Akin, S. , Altuner, Z. , Polat, F. , & Dal, T. (2018). Spatial and temporal variation in food web structure of an impounded river in Anatolia. Marine and Freshwater Research, 69, 1453–1471. 10.1071/MF17270

[ece38304-bib-0019] Lau, D. C. P. , Leung, K. M. Y. , & Dudgeon, D. (2009). What does stable isotope analysis reveal about trophic relationships and the relative importance of allochthonous and autochthonous resources in tropical streams? A synthetic study from Hong Kong. Freshwater Biology, 54, 127–141. 10.1111/j.1365-2427.2008.02099.x

[ece38304-bib-0020] Lewis, W. M. , Hamilton, S. K. , Rodríguez, M. A. , Saunders, J. F. , & Lasi, M. A. (2001). Foodweb analysis of the Orinoco floodplain based on production estimates and stable isotope data. Journal of the North American Benthological Society, 20, 241–254. 10.2307/1468319

[ece38304-bib-0021] March, J. G. , & Pringle, C. M. (2003). Food web structure and basal resource utilization along a tropical island stream continuum, Puerto Rico. Biotropica, 35, 84–93. 10.1111/j.1744-7429.2003.tb00265.x

[ece38304-bib-0022] Medeiros, E. S. , & Arthington, A. H. (2011). Allochthonous and autochthonous carbon sources for fish in floodplain lagoons of an Australian dryland river. Environmental Biology of Fishes, 90, 1–17. 10.1007/s10641-010-9706-x

[ece38304-bib-0046] Morse, J. C. , Yang, L.‐F. , & Tian, L.‐X. (1994). Aquatic insects of China useful for monitoring water quality. Hohai University Press.

[ece38304-bib-0023] Neres‐Lima, V. , Brito, E. F. , Krsulović, F. A. , Detweiler, A. M. , Hershey, A. E. , & Moulton, T. P. (2016). High importance of autochthonous basal food source for the food web of a Brazilian tropical stream regardless of shading. International Review of Hydrobiology, 101, 132–142. 10.1002/iroh.201601851

[ece38304-bib-0024] Parnell, A. C. , Inger, R. , Bearhop, S. , & Jackson, A. L. (2010). Source partitioning using stable isotopes: Coping with too much variation. PLoS One, 5, e9672. 10.1371/journal.pone.0009672 20300637 PMC2837382

[ece38304-bib-0025] Peterson, B. J. , & Fry, B. (1987). Stable isotopes in ecosystem studies. Annual Review of Ecology and Systematics, 18, 293–320. 10.1146/annurev.es.18.110187.001453

[ece38304-bib-0026] Pingram, M. A. , Collier, K. J. , Hamilton, D. P. , David, B. O. , & Hicks, B. J. (2012a). Carbon sources supporting large river food webs: A review of ecological theories and evidence from stable isotopes. Freshwater Reviews, 5, 85–104. 10.1608/FRJ-5.2.476

[ece38304-bib-0027] Pingram, M. A. , Collier, K. J. , Hamilton, D. P. , Hicks, B. J. , & David, B. O. (2012b). Spatial and temporal patterns of carbon flow in a temperate, large river food web. Hydrobiologia, 729, 107–131. 10.1007/s10750-012-1408-2

[ece38304-bib-0028] Post, D. M. , Layman, C. A. , Arrington, D. A. , Takimoto, G. , Quattrochi, J. , & Montaña, C. G. (2007). Getting to the fat of the matter: Models, methods and assumptions for dealing with lipids in stable isotope analyses. Oecologia, 152, 179–189. 10.1007/s00442-006-0630-x 17225157

[ece38304-bib-0029] Rounick, J. , & Hicks, B. J. (1985). The stable carbon isotope ratios of fish and their invertebrate prey in four New Zealand rivers. Freshwater Biology, 15, 207–214. 10.1111/j.1365-2427.1985.tb00193.x

[ece38304-bib-0031] Thorp, J. H. , & Delong, M. D. (1994). The riverine productivity model: An heuristic view of carbon sources and organic processing in large river ecosystems. Oikos, 70, 305–308. 10.2307/3545642

[ece38304-bib-0032] Thorp, J. H. , & Delong, M. D. (2002). Dominance of autochthonous autotrophic carbon in food webs of heterotrophic rivers. Oikos, 96, 543–550. 10.1034/j.1600-0706.2002.960315.x

[ece38304-bib-0033] Thorp, J. H. , Delong, M. D. , Greenwood, K. S. , & Casper, A. F. (1998). Isotopic analysis of three food web theories in constricted and floodplain regions of a large river. Oecologia, 117, 551–563. 10.1007/s004420050692 28307681

[ece38304-bib-0034] Thorp, J. H. , Thoms, M. C. , & Delong, M. D. (2006). The riverine ecosystem synthesis: Biocomplexity in river networks across space and time. River Research and Applications, 22, 123–147. 10.1002/rra.901

[ece38304-bib-0035] Vannote, R. L. , Minshall, G. W. , Cummins, K. W. , Sedell, J. R. , & Cushing, C. E. (1980). The river continuum concept. Canadian Journal of Fisheries and Aquatic Sciences, 37, 130–137. 10.1139/f80-017

[ece38304-bib-0039] Wang, S. , Wang, L. , Chang, H.‐Y. , Li, F. , Tang, J.‐P. , Zhou, X.‐A. , Li, X. , Tian, S.‐M. , Lin, H.‐J. , & Yang, Y. (2018a). Longitudinal variation in energy flow networks along a large subtropical river, China. Ecological Modelling, 387, 83–95. 10.1016/j.ecolmodel.2018.08.019

[ece38304-bib-0040] Wang, S. , Wang, T.‐T. , Tang, J.‐P. , Wang, L. , Yang, Y. , Lin, H.‐J. , Chang, H.‐Y. , Zhou, X.‐A. , Li, X. , & Wang, M. (2018b). Longitudinal variation in fish prey utilization, trophic guilds, and indicator species along a large subtropical river, China. Ecology and Evolution, 8, 11467–11483. 10.1002/ece3.4577 30598749 PMC6303697

[ece38304-bib-0038] Wang, S. , Tang, J.‐P. , Su, L.‐H. , Fan, J.‐J. , Chang, H.‐Y. , Wang, T.‐T. , Wang, L. , Lin, H.‐J. , & Yang, Y. (2019). Fish feeding groups, food selectivity, and diet shifts associated with environmental factors and prey availability along a large subtropical river, China. Aquatic Sciences, 81, 31. 10.1007/s00027-019-0628-1

[ece38304-bib-0036] Wang, S. , Luo, B.‐K. , Qin, Y.‐J. , Su, L.‐H. , Stewart, S. D. , Wang, T.‐T. , Tang, J.‐P. , He, B.‐D. , Zhang, J.‐H. , Lin, H.‐J. , & Yang, Y. (2020a). Consumer‐diet discrimination of *δ* ^13^C and *δ* ^15^N: Source‐ and feeding‐oriented patterns based on gut content analysis in a large subtropical river of China. River Research and Applications, 36, 1124–1136.

[ece38304-bib-0037] Wang, S. , Su, L.‐H. , Luo, B.‐K. , Qin, Y.‐J. , Stewart, S. D. , Tang, J.‐P. , Wang, T.‐T. , Yang, Y. , & Cheng, G. (2020b). Stable isotopes reveal effects of natural drivers and anthropogenic pressures on isotopic niches of invertebrate communities in a large subtropical river of China. Environmental Science and Pollution Research, 27, 36132–36146. 10.1007/s11356-020-09252-8 32557028

[ece38304-bib-0045] Wang, S. , Luo, B.‐K. , Qin, Y.‐J. , Zhao, J.‐G. , Wang, T.‐T. , Stewart, S. D. , Yang, Y. , Chen, Z.‐B. , & Qiu, H.‐X. (2020c). Fish isotopic niches associated with environmental indicators and human disturbance along a disturbed large subtropical river in China. Science of The Total Environment, 750, 141667. 10.1016/j.scitotenv.2020.141667 32871370

[ece38304-bib-0044] Wang, S. , Wang, T.‐T. , Lin, H.‐J. , Stewart, S. D. , Cheng, G. , Li, W. , Yang, F.‐J. , Huang, W.‐D. , Chen, Z.‐B. , & Xie, S.‐G. (2021). Impacts of environmental factors on the food web structure, energy flows, and system attributes along a subtropical urban river in southern China. Science of The Total Environment, 794, 148673. 10.1016/j.scitotenv.2021.148673 34217084

[ece38304-bib-0041] Welcomme, R. L. , Winemiller, K. O. , & Cowx, I. G. (2006). Fish environmental guilds as a tool for assessment of ecological condition of rivers. River Research and Applications, 22, 377–396. 10.1002/rra.914

[ece38304-bib-0042] Wu, Y. (2016). Periphyton: Functions and application in environmental remediation. Elsevier.

[ece38304-bib-0043] Zeni, J. O. , & Casatti, L. (2014). The influence of habitat homogenization on the trophic structure of fish fauna in tropical streams. Hydrobiologia, 726, 259–270. 10.1007/s10750-013-1772-6

