# Supplementary material for: Longitudinal pattern of resource utilization by aquatic consumers along a disturbed subtropical urban river: Estimating the relative contribution of resources with stable isotope analysis
Source: Ecol Evol. 2021 Nov 11;11(23):16763–75. doi: 10.1002/ece3.8304 (PMC8668758; doi:10.1002/ece3.8304)
Supplement: Supplementary file 1 — Supplementary Material [file ECE3-11-16763-s001.docx]

**SUPPORTING INFORMATION**

**Supplementary methods for collecting fish, invertebrates, and basal organic matter resources**

Electrofishing equipment consisted of a 24-kW generator, a 12V-160A lithium battery, a silicon-controlled inverter, and two continuously adjustable voltage and frequency regulators. A copper probe cathode was installed on one 1-4 m-long telescopic insulated rod, and a 20 cm-diameter ring anode with a 3 mm-mesh net was installed on another, identical rod. This equipment was used to effectively stun and collect fish (individual weight < 10 kg) in a 2 m wide × 2 m long × 3.5 m deep water column. Due to varying water levels, two electrofishing operations were conducted as follows: 1) At wadeable sites, single-pass backpack electrofishing was performed simultaneously by two operators moving in a zig-zag pattern. The electrofishing equipment was adjusted to a low voltage and a mixed frequency, and the walking speed was controlled to ensure a sampling effort of approximately 8 m^2^ min^-1^ over 30 minutes; 2) At non-wadeable sites, a 6-m-long welded diesel powered hull boat was used for boat-electrofishing, and a bamboo quant was used to propel the boat to eliminate noise disturbance to fish. Electrofishing equipment was adjusted at a high voltage and main frequency, and the paddling speed was controlled to ensure a sampling effort of approximately 6 m^2^ min^-1^. Fish biomass per unit area was calculated as the wet weight of the sampled fish specimens divided by the effective sampling area (i.e., electrofishing efforts × sampling time), which was expressed in grams/m^2^.

Two sampling methods for invertebrates, plant debris, and submerged hydrophytes are as follows: 1) At sites 1-3 in the mountain streams, with cobble/gravel beds, the substrates were washed and sieved with a kick-net with 500 μm mm-mesh, with instream sampling areas of 1-5 m^2^. 2) At sites 4-8 in the middle and lower reaches with large water depths and sand beds, a Petersen grab bucket sampler was used, with a sampling area of 0.04 m^2^ and 25 grab replicates taken across the river section. At each sampling site, plant debris and submerged hydrophytes were separated and weighed in the field, and the remaining invertebrates were collected and subsequently identified in the laboratory to at least family level.

Periphyton, including epilithic diatoms, vascular plant detritus, and aquatic invertebrates attached to the substrates (e.g., cobbles and rocks), were scraped with a toothbrush. Pure algae cells were separated from the detritus and microinvertebrates using density fractionation in colloidal silica. Under 40-100 × magnification, the epilithic algae were typically dominated by diatoms. Leaves from approximately 10 individuals of the dominant submerged and riparian vascular plant species were collected by hand. Extraneous materials, e.g., periphyton and mud, were carefully scraped and washed from the leaf surfaces with de-ionized water before freeze-drying.

To sample suspended particulate organic matter (SPOM, 0.45~112 μm), 2-5 L of water was collected using a cascade filtering device with two piled plankton nets (mesh size 112 μm) to eliminate large zooplankton and detrital particles and filtered through pre-combusted (450 °C for 4 h) Whatman GF⁄F filters (*n* = 6). Visible detrital particles were removed before freeze-drying the samples for storage.

**Table S1** Habitat characteristics and geographic features (averaged for rainy and dry seasons) at the 8 sampling sites along the Liuxi River

|  | S1 | S2 | S3 | S4 | S5 | S6 | S7 | S8 |
| --- | --- | --- | --- | --- | --- | --- | --- | --- |
| Latitude | 23°54'54.9" | 23°48'50.3″ | 23°43'37.2″ | 23°39'28.5″ | 23°30'2.6″ | 23°25'5.1″ | 23°20'22.4″ | 23°14'35.4" |
| Longitude | 113°53'47.2" | 113°50'16.9″ | 113°43'56.4″ | 113°40'43.2″ | 113°32'12.9″ | 113°26'42.0″ | 113°18'24.1″ | 113°12'57.1" |
| Elevation (m) | 236.0 | 183.3 | 62.4 | 37.6 | 23.1 | 8.5 | -1.0 | -2.8 |
| Distance to estuary (km) | 103.2 | 81.6 | 68.6 | 54.2 | 41.9 | 28.8 | 16.9 | 3.2 |
| Slope (^○^) | 32.5 | 18.4 | 13.6 | 8.1 | 4.8 | 1.1 | 0.6 | 0.1 |
| Wetted stream width (m) | 3.9 | 19.9 | 32.7 | 58.5 | 64.2 | 149.0 | 173.1 | 232.9 |
| Flow discharge (m^3^/s) | 0.43 | 5.74 | 20.9 | 58.0 | 80.9 | 103.2 | 143.9 | 197.3 |
| Riffle habitat (% of habitat area) | 96.2 | 53.2 | 18.3 | 6.9 | - | - | - | - |
| Pool habitat (% of habitat area) | 3.8 | 18.4 | 6.1 | 0.8 | - | - | - | - |
| Canopy cover (%) | 92.3 | 43.2 | 33.9 | 20.7 | 10.4 | <1.0 | <1.0 | <1.0 |
| Seasonal floodplain area (m^2^) | 80.8 | 32.8 | 43.7 | 18.4 | 13.2 | - | - | - |
| Riparian buffer width (m) | 23.7 | 8.9 | 4.3 | 3.2 | <1.0 | - | - | - |
| Gravel-cobble substrate (% of substrate area) | 87.3 | 23.2 | 20.3 | 19.3 | 16.5 | 12.6 | - | - |
| Silt-sand substrate (% of substrate area) | 6.87 | 35.4 | 40.5 | 49.0 | 65.1 | 87.4 | 100 | 100 |
| Urban land (% of land area) | <0.1 | 21.4 | 30.9 | 42.1 | 53.1 | 65.8 | 80.4 | 90.1 |
| Agricultural land (% of land area) | 5.43 | 43.2 | 54.9 | 20.8 | 28.7 | 20.6 | 10.4 | 6.9 |
| Forestry land (% of land area) | 76.5 | 17.7 | 7.1 | 18.6 | 9.1 | 6.8 | 4.6 | 1.5 |
| Riparian vegetation coverage (%) | 96.7 | 53.8 | 43.2 | 37.9 | 30.2 | 15.6 | 7.4 | <1.0 |
| Nearshore concrete revetment (km) | <0.1 | - | 1.4 | 2.6 | 4.3 | 5.3 | 6.9 | 8.7 |
| Nearshore population (thousand) | <0.5 | 11.6 | 23.1 | 30.5 | 64.1 | 42.1 | 31.6 | 101.5 |
| Pollution sources | - | - | - | + | + | + | + | + |
| Dams | - | + | - | - | + | - | + | - |
| Substrate type | Cobbles | Boulders/Cobbles/Coarse gravel | | Gravel/Coarse sand | | Sand | Sand and silt | |
| Vegetation type | Riparian shrubs and herbage | Upland eucalyptus and bamboo forest; submerged tape grass | | Riparian b[amboo](app:ds:bamboo), arista, reeds, herbage;  floating and submerged watermilfoil | | | Riparian reeds and herbage | |

Absence (-)

**Table S2** Physical and chemical parameters of water quality (averaged for rainy and dry seasons) at the 8 sampling sites along the Liuxi River

| Parameters | S1 | S2 | S3 | S4 | S5 | S6 | S7 | S8 |
| --- | --- | --- | --- | --- | --- | --- | --- | --- |
| WT (^o^C) | 20.60 | 20.67 | 22.59 | 20.95 | 22.36 | 25.96 | 24.68 | 19.64 |
| pH | 7.53 | 7.48 | 7.22 | 7.18 | 7.02 | 6.86 | 6.97 | 7.19 |
| DO (mg/L) | 7.28 | 7.12 | 7.20 | 6.86 | 7.00 | 5.28 | 6.60 | 0.74 |
| Depth (m) | 0.148 | 0.264 | 0.635 | 0.989 | 1.251 | 1.601 | 1.765 | 1.959 |
| Current velocity (m/s) | 0.462 | 0.375 | 0.149 | 0.115 | 0.110 | 0.102 | 0.094 | 0.083 |
| EC (μS/cm) | 27.84 | 54.03 | 19.79 | 63.05 | 36.57 | 112.33 | 37.73 | 278.88 |
| TSS (mg/L) | 6.56 | 10.38 | 22.02 | 13.77 | 12.61 | 25.12 | 16.98 | 15.62 |
| PPI (mg/L) | 0.962 | 1.067 | 0.951 | 2.299 | 1.436 | 1.659 | 3.376 | 4.996 |
| COD (mg/L) | 0.137 | 0.178 | 1.019 | 2.765 | 6.683 | 7.353 | 11.834 | 20.564 |
| BOD_5_ (mg/L) | 1.080 | 1.237 | 1.466 | 2.040 | 2.172 | 1.676 | 2.611 | 4.765 |
| NH_3_-N (mg/L) | 0.063 | 0.072 | 0.175 | 0.879 | 0.486 | 0.807 | 2.648 | 5.823 |
| NO_X_^－^-N (mg/L) | 0.161 | 0.205 | 0.344 | 2.121 | 0.771 | 2.269 | 4.447 | 10.748 |
| TP (mg/L) | 0.012 | 0.016 | 0.036 | 0.216 | 0.131 | 0.113 | 0.181 | 0.782 |
| Chl-*a* (mg/L) | 0.720 | 0.635 | 1.815 | 3.240 | 7.182 | 4.389 | 8.745 | 2.837 |
| [Se (mg/L)](javascript:;) | 0.0004 | 0.0002 | 0.0005 | 0.0016 | 0.0018 | 0.0008 | 0.0020 | 0.0006 |
| Cd (mg/L) | - | - | - | 0.0002 | 0.0005 | - | 0.0004 | 0.0001 |
| Cr^6+^ (mg/L) | 0.0014 | 0.0020 | 0.0025 | 0.0040 | 0.0036 | 0.0043 | 0.0050 | 0.0037 |
| Pb (mg/L) | 0.0040 | 0.0051 | 0.0084 | 0.0095 | 0.0101 | 0.0101 | 0.0095 | 0.0149 |
| Cu (mg/L) | 0.0002 | 0.0003 | 0.0024 | 0.0038 | 0.0024 | 0.0044 | 0.0028 | 0.0027 |
| Zn (mg/L) | 0.0024 | 0.0049 | 0.0024 | 0.0237 | 0.0067 | 0.0028 | 0.0294 | 0.0383 |
| As (mg/L) | - | - | 0.0003 | 0.0001 | 0.0003 | 0.0018 | 0.0019 | 0.0028 |
| Hg (mg/L) | - | - | - | - | - | - | 0.0001 | 0.0000 |
| ASs (mg/L) | 0.0014 | 0.0023 | 0.0146 | 0.0447 | 0.0427 | 0.0175 | 0.0595 | 0.0243 |
| Petroleum (mg/L) | 0.0049 | 0.0056 | 0.0060 | 0.0178 | 0.0424 | 0.0808 | 0.0990 | 0.1951 |
| Volatile phenol (mg/L) | - | 0.0001 | 0.0003 | 0.0006 | 0.0005 | 0.0006 | 0.0009 | 0.0013 |
| Sulfides (mg/L) | 0.0014 | 0.0021 | 0.0034 | 0.0062 | 0.0069 | 0.0084 | 0.0101 | 0.0207 |
| Fluorides (mg/L) | 0.0631 | 0.0309 | 0.0982 | 0.1013 | 0.2673 | 0.2221 | 0.2860 | 0.2923 |
| Cyanides (mg/L) | - | 0.0004 | 0.0005 | 0.0007 | 0.0008 | 0.0011 | 0.0016 | 0.0020 |
| Faecal Coliform (cells/L) | 197 | 301 | 2043 | 3745 | 4474 | 9602 | 111563 | 206653 |

WT, water temperature; DO, dissolved oxygen; EC, electrical conductivity; PPI, potassium permanganate index; TSS, total suspended solids; COD, chemical oxygen demand; BOD_5_, five-days biochemical oxygen demand; NH_3_-N, ammonia nitrogen; NO_X_^－^-N, nitrate nitrogen = NO_3_^－^-N + NO_2_^－^-N; TP, total phosphorus; Chl-*a*, chlorophyll *a*; ASs, anionic surfactants.

**Table S3** The dominant species/genus/family composition of fish and invertebrate functional feeding groups (FFGs) used for stable isotope measurement. The ages of all fish samples are > 1.5 years old. BL: body length (cm); BW: body width (cm); *n*: individual number collected in each replicate sample; *N*: the sample size of stable isotope measurement for each FFG at each site; c-g: collector-gatherer.

| FFGs | Taxon  Species/Genus/Family | Sampling sites | Individual size | *n* for each sample | *N* at each site |
| --- | --- | --- | --- | --- | --- |
| Fish omnivore | *Micronemacheilus puicher* | #1 | 8.3 > BL > 5.2 cm | 1 | 5 |
|  | *Cobitis sinensis* | #2 − #4 | 7.2 > BL > 5.1 cm | 1 | 6 |
|  | *Acrossocheilus parallens* | #5 − #7 | 7.8 > BL > 10 cm | 1 | 6−7 |
|  | *Carassioides auratus* | #8 | 17 > BL > 12 cm | 1 | 5 |
| Fish periphytivore | *Bufo bankorensis* (tadpole) | #1 | 2.6 > BL > 1.5 cm | 1 | 5 |
|  | *Pseudogastromyzon changtingensis* | #2 | 8.2 > BL > 5.1 cm | 1 | 5 |
|  | *Vanmanenia caldwelli* | #3 | 6.9 > BL > 5.3 cm | 1 | 5 |
|  | *Hemiculter leucisculus* | #4 − #8 | 12 > BL > 7.3 cm | 1 | 5 |
| Fish insectivore | *Rhinogobius giurinus* | #2 − #5 | 8.8 > BL > 5.2 cm | 1 | 5−6 |
| Fish herbivore | *Ctenopharyngodon idellus* | #6 | 18 > BL > 12 cm | 1 | 5 |
| Fish annelidivore | *Macropodus opercularis* | #8 | 13 > BL > 7.1 cm | 1 | 5 |
| Fish invertivore | *Hemibarbus medius* | #3 − #5 | 15 > BL > 8.4 cm | 1 | 5−6 |
| Fish molluscivore | *Sarcocheilichthys nigripinnis* | #3 − #5 | 13 > BL > 7.9 cm | 1 | 5 |
|  | *Squaliobarbus curriculus* | #6 − #8 | 14 > BL > 9.1 cm | 1 | 5−6 |
| Fish crustaceavore | *Silurus asotus* | #3 | 16 > BL > 12 cm | 1 | 5 |
|  | *Silurus cochinchinensis* | #4 | 19 > BL > 13 cm | 1 | 5 |
|  | *Mastacembelus armatus* | #5 − #6 | 16 > BL > 12 cm | 1 | 5 |
|  | *Pelteobagrus fulvidraco* | #8 | 17 > BL > 13 cm | 1 | 5 |
| Fish piscivore | *Channa asiatica* | #4 | 17 > BL > 12 cm | 1 | 5 |
|  | *Channa maculata* | #5 − #6 | 21 > BL > 15 cm | 1 | 6 |
|  | *Clarias fuscus* | #7 − #8 | 16 > BL > 10 cm | 1 | 6 |
| Fish detritivore | *Misgurnus anguillicaudatus* | #4 − #7 | 12 > BL > 7.2 cm | 1 | 6−7 |
|  | *Paramisgurnus dabryanus* | #8 | 14 > BL > 7.3 cm | 1 | 5 |
| Fish phytoplanktivore | *Hypophthalmichthys nobilis* | #6 − #8 | 25 > BL > 17 cm | 1 | 5 |
| Fish zooplanktivore | *Protosalanx hyalocranius* | #6 | 14 > BL > 6.9 cm | 1 | 5 |
|  | *Coilia grayii* | #7 − #8 | 7.8 > BL > 5.2 cm | 1 | 8 |
| Insect collector-gatherer | Chironomidae (Stenochironomus and Orthocladiinae) | #1 − #6 | 1.3 > BL > 0.3 cm | ≥ 40 | 7−8 |
| Insect collector-filterer | Polygonidae | #1 − #3 | 1.4 > BL > 0.6 cm | ≥ 12 | 5 |
| Insect scraper | Heptageniidae | #1 − #3 | 1.0 > BL > 0.3 cm | ≥ 18 | 5 |
| Insect c-g/scraper | Baetidae | #1 − #3 | 0.9 >BL > 0.3 cm | ≥ 22 | 5 |
| Insect shredder | Haliplidae | #2 − #3 | 0.6 > BL > 0.3 cm | ≥ 24 | 5 |
| Insect predator | Gomphidae | #1 − #7 | 3.5 > BL > 1.8 cm | 1 | 8−9 |
| Gastropod scraper | *Melania lilbertina* | #1 − #2 | 2.5 > BL > 1.2 cm | 1 | 5 |
|  | *Bellamya purificata* | #3 − #8 | 2.8 > BL > 1.4 cm | 1 | 5−6 |
| Bivalve filterer | *Corbicula fluminea* | #1 − #7 | 2.2 > BW > 1.0 cm | ≥ 2 | 5−7 |
|  | *Limnoperna lacustris* | #8 | 1.6 > BL > 0.5 cm | ≥ 4 | 5 |
| Shrimp scraper | *Caridina nilotica gracilipes* | #3 − #6 | 1.5 > BL > 0.7 cm | ≥ 5 | 6−7 |
|  | *Neocaridina denticulata sinensis* | #7 − #8 | 2.2 > BL > 1.0 cm | ≥ 3 | 7 |
| Shrimp collector | *Macrobranchium nipponense* | #3 − #8 | 6.4 > BL > 2.8 cm | 1 | 5−6 |
| Shrimp predator | *Oratosquilla oratoria* | #8 | 12 > BL > 4.5 cm | 1 | 5 |
| Shrimp filterer | *Parapenaeopsis cultrirostris* | #8 | 5.4 > BL > 2.8 cm | 1 | 5 |
| Crab collector | *Chiromantes dehaani* | #4 − #8 | 7.6 > BW > 2.8 cm | 1 | 5−7 |
| Zooplankton | Copepoda and nauplius larva of Malacostraca | #4 − #8 | Regardless of size | ≥ 200 | 7−8 |
| Oligochaete collector | *Limnodrilus* sp. | #7 | 5.2 > BL > 2.0 cm | ≥ 10 | 5 |
| Polychaete collector | Nereidae sp. | #8 | 8.4 > BL > 5.2 cm | 1 | 5 |

**
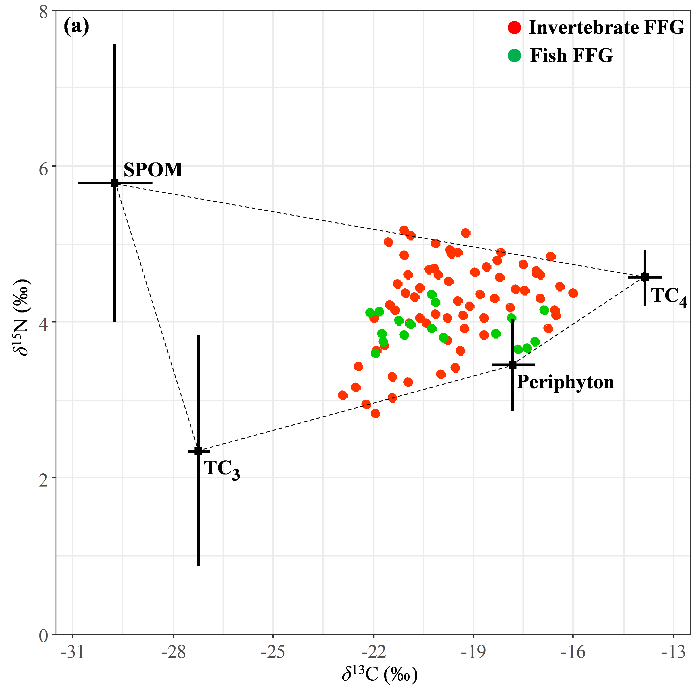

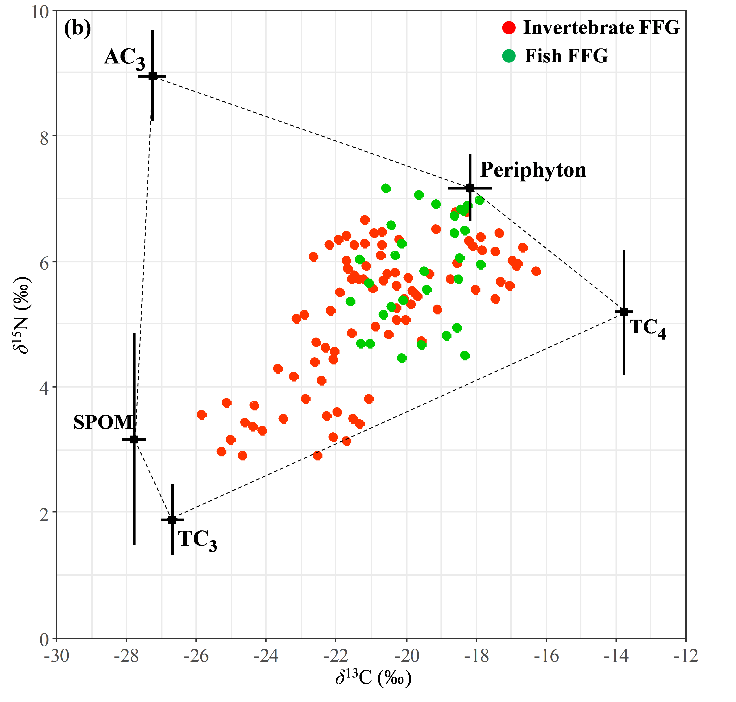

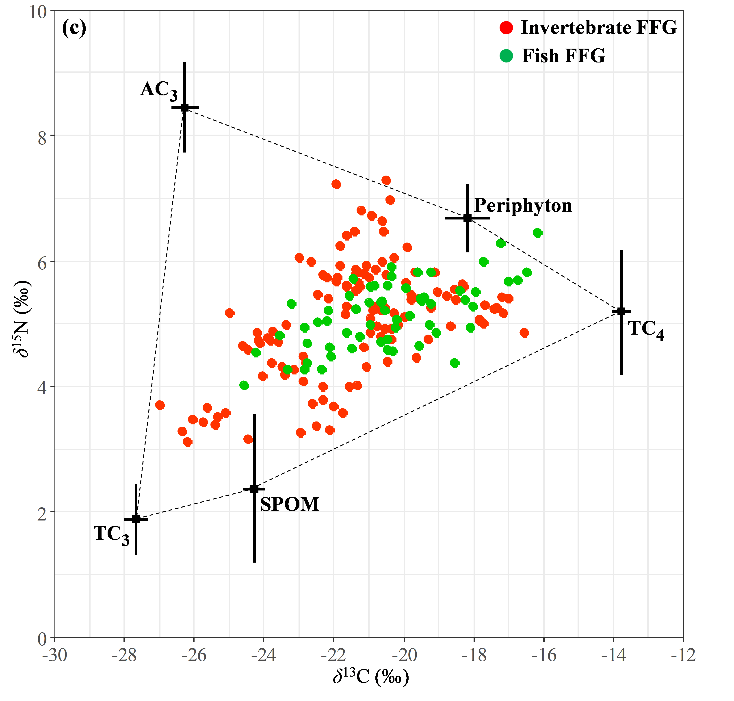

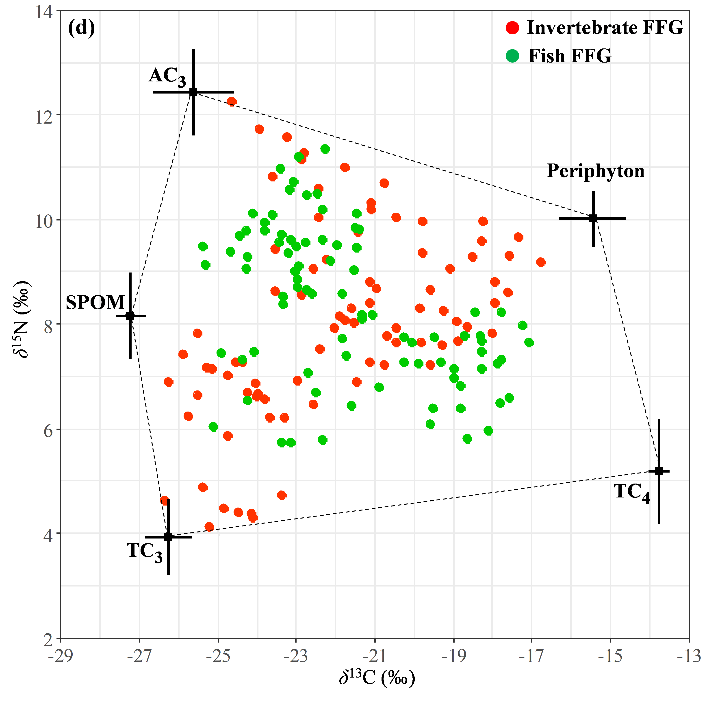

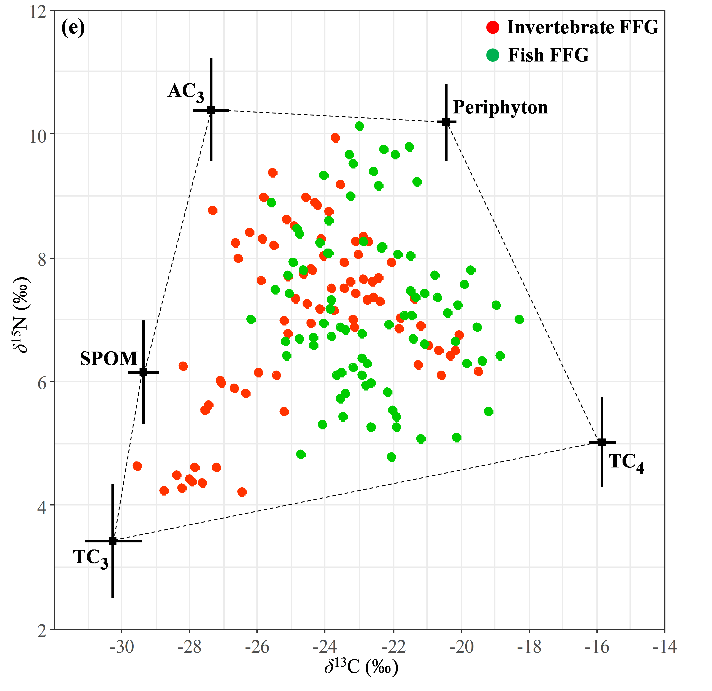

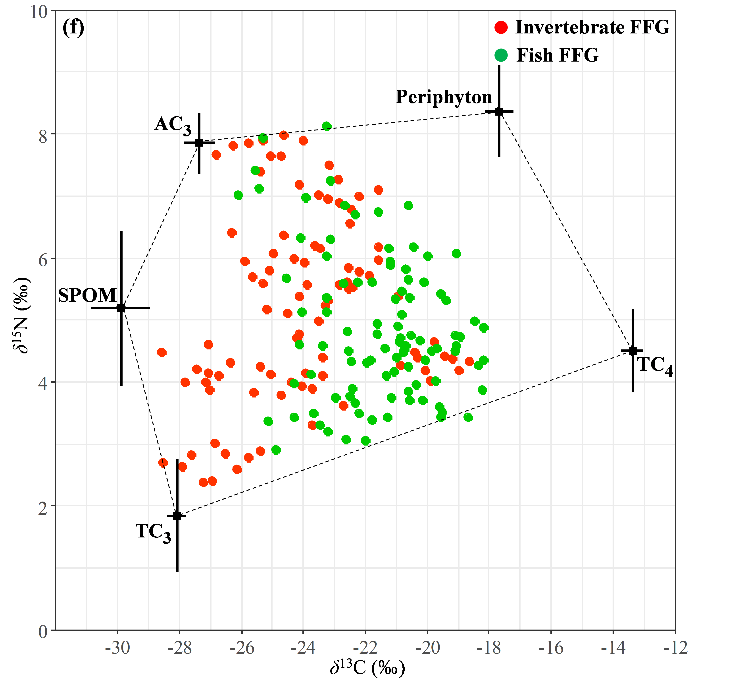

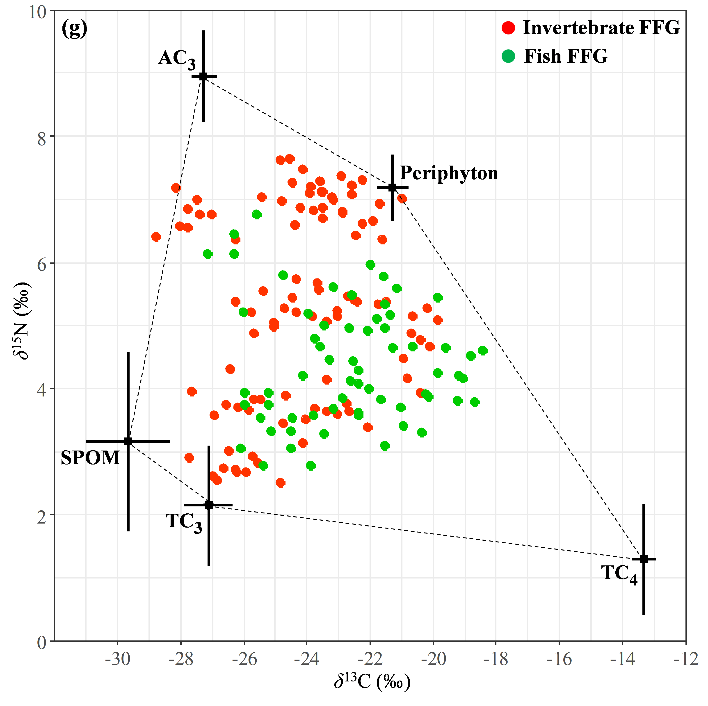

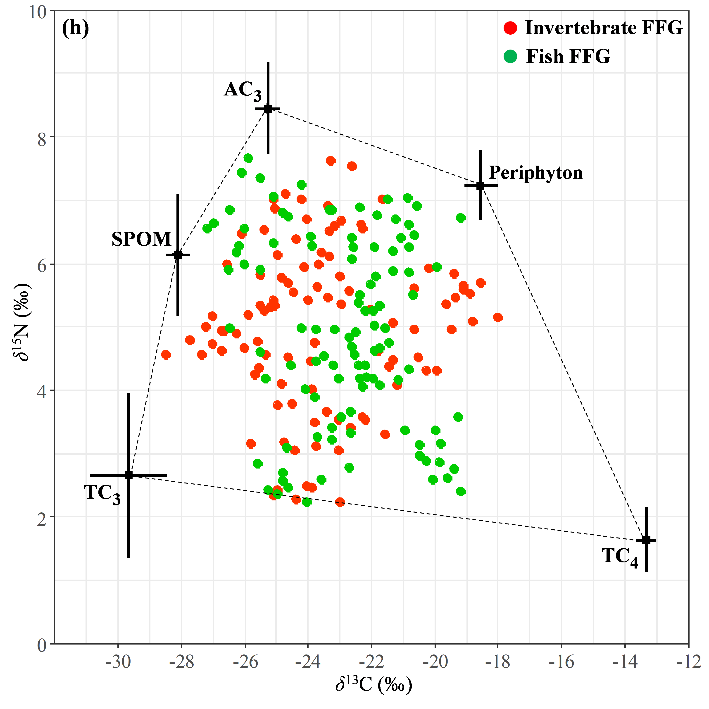
**

**Figure S1** *δ*^13^C-*δ*^15^N biplot of consumer functional feeding groups (FFGs) and five basal resources at sampling sites 1-8 along the Liuxi River. The convex hull at each site was occupied by the mean *δ*^13^C and *δ*^15^N values of local basal resources. (a) Site 1, (b) Site 2, (c) Site 3, (d) Site 4, (e) Site 5, (f) Site 6, (g) Site 7, and (h) Site 8. SPOM, suspended particulate organic matter; AC_3_, aquatic C_3_ plants; TC_3_, terrestrial C_3_ plants; TC_4_, terrestrial C_4_ plants.
